# Texturization of a Blend of Pea and Destarched Oat Protein Using High-Moisture Extrusion

**DOI:** 10.3390/foods10071517

**Published:** 2021-07-01

**Authors:** Mika Immonen, Angga Chandrakusuma, Juhani Sibakov, Minna Poikelispää, Tuula Sontag-Strohm

**Affiliations:** 1 Department of Food and Nutrition, Faculty of Agriculture and Forestry, University of Helsinki, P.O. Box 27, FI-00014 Helsinki, Finland; Tuula.sontag-strohm@helsinki.fi; 2 Valio Ltd., P.O. Box 10, FI-00039 Helsinki, Finland; Angga.Chandrakusuma@valio.fi; 3 Fazer Bakery Finland, P.O. Box 17, FI-00941 Helsinki, Finland; Juhani.sibakov@fazer.com; 4 Department of Materials Science, Faculty of Engineering Sciences, Tampere University, P.O. Box 689, FI-33014 Tampere, Finland; Minna.poikelispaa@tuni.fi

**Keywords:** closed cavity rheometer, destarched oat protein concentrate, high-moisture extrusion

## Abstract

Grain protein fractions have great potential as ingredients that contain high amounts of valuable nutritional components. The aim of this study was to study the rheological behavior of destarched oat and pea proteins and their blends in extrusion-like conditions with a closed cavity rheometer. Additionally, the possibility of producing fibrous structures with high-moisture extrusion from a blend of destarched oat and pea protein was investigated. In the temperature sweep measurement (60–160 °C) of the destarched oat protein concentrate and pea protein isolate blend, three denaturation and polymerization sections were observed. In addition, polymerization as a function of time was recorded in the time sweep measurements. The melting temperature of grain proteins was an important factor when producing texturized structures with a high-moisture extrusion. The formation of fibrillar structures was investigated with high-moisture extrusion from the destarched oat and pea protein blend at temperatures ranging from 140 to 170 °C. The protein–protein interactions were significantly influenced in the extruded samples. This was due to a decrease in the amount of extractable protein in selective buffers. In particular, there was a decrease in non-covalent and covalent bonds due to the formation of insoluble protein complexes.

## 1. Introduction

In recent years, heightened concerns over the sustainability of animal protein production and the influence of red and processed meat on health have boosted the demand for plant-based high-protein foods [1]. According to Afshin et al. [2], the global consumption of processed meat is 90% higher and the intake of red meat is 18% higher than that recommended. One way of limiting meat consumption is by offering tasty, easy-to-use plant-based alternatives to replace animal protein products [3]. Traditional plant-based foods that have been used as meat replacers include tofu and tempeh, which are produced from soybean or fava beans, and seitan made from wheat gluten [4,5,6]. Modern meat analogs, such as sausages or patties, are generally made from soy or pea protein [7].

Usually, these types of meat-analog products are made from texturized vegetable protein (usually soy) obtained through low-moisture extrusion using a combination of hydrocolloids, such as carrageenan and methylcellulose [8,9].

Extrusion technology can be utilized to create fibrous products from plant proteins that have high moisture content (40–80%) and fibrous, non-expanded textures [10]. Studies have demonstrated that similar fibrous structures can be made from soy and pea protein isolates as well as wheat gluten [11,12,13]. In addition, oat and pea protein blends have been used to produce fibrillar structures using a twin-screw extruder without cooling die [14,15]. For example, De angelis et al. [15] produced meat analogs from an oat and pea protein isolate blend (ratio 30:70 *w/w*%) using low-moisture extrusion with parameters such as a screw speed of 600 rpm and an extrusion temperature of 150 °C.

During extrusion, plant-based proteins are modified by pressure, thermal and mechanical forces. The barrel of the extruder is used in heating and maintaining the temperature whilst the shear of the screws leads to mechanical and thermal forces [11]. The extrusion process can result in the denaturation of proteins as well as changes in the molecular structure, which can lead to the formation of protein aggregates. Additionally, proteins undergo unfolding and cross-linking during the extrusion, which leads to the formation of protein–protein and protein–non-protein polymers with large molecular weights [16,17]. To generate a fibrous structure, a long cooling die is required at the end of the extruder in order to promote the reorientation in the flow direction to form an anisotropic protein network [18]. Furthermore, the cooling die prevents the extruded material from expanding or puffing, which often happens in low-moisture extrusion.

The extrusion process is described as a “black box” due to a poor understanding of what happens to the proteins within the barrel [10]. One method to study the behavior of proteins in extrusion-like conditions is a closed cavity rheometer, used in the rubber industry to study the melting properties of polymers [19]. The closed cavity rheometer combines the advantages of conventional rheometers with high shear cells and therefore can be used to study biopolymeric melts. Similar treatments to extrusion (i.e., high temperature and high mechanical torque) can be applied directly to the studied sample at elevated pressure and without evaporation of water. The closed cavity rheometer has been used to define the behavior of plant-based proteins such as wheat gluten [19], rapeseed [20], pea and soy proteins [21]. Despite a great number of studies on high-moisture extrusion, there are not many scientific studies on the “melting” properties of oat proteins or oat proteins mixed with other grain legume protein sources, such as pea.

The aim of this study was to examine the behavior of oat and pea proteins and their blend in extrusion-like conditions with a closed cavity rheometer. Additionally, fibrous structures formed by high-moisture extrusion from a blend of destarched oat and pea protein were investigated.

## 2. Materials and Methods

### 2.1. Materials

Commercial pea protein isolate (PPI) was obtained from Roquette (Nutralys F85M, Roquette, Lestrem, France). Commercial wheat gluten (WG) was obtained from Kröner Stärke (Vital Wheat Gluten, Kröner-96 Stärke, Ibbenbüren, Germany). Oat protein concentrate (OPC) was kindly provided by Fazer Mills (Lahti, Finland). Thermostable bacterial α-amylase Gamalpha Spezial (activity according to specifications was 200,000 TAU/g) was obtained from AB-enzymes (Darmstadt, Germany).

### 2.2. Preparation of Destarched OPC

The oat protein concentrate (30% *w/w*) was mixed with water (70% *w/w*) and 1% of the total volume of α-amylase was mixed with water before adding the oat protein concentrate. The oat–water suspension was heated in a drying oven (Memmert GmbH, Buechenbach, Germany) at 80 °C for 15 min, and the suspension was mixed by shaking it vigorously by hand in five-minute intervals. Heat-treatment of the suspension was continued in a dry cabinet at 50 °C for 30 min under continuous mixing. This was done to completely homogenizing the suspension and to allow the further hydrolysis of starch. The hydrolyzation of starch was determined visually in the drop of viscosity. The suspensions were centrifuged (Beckman, Avanti J25l, Palo Alto, CA, USA) (9000 rpm, 14,335× *g*; 10 min; 20 °C). After centrifugation, the supernatants were discarded and the precipitates were dried in a cabinet vegetable dryer (Orakas, Lemi, Finland) at 50 °C for 6 h. The dried precipitates were then milled to a fine powder with a centrifugal mill (Retsch, Haan, Germany) using 8000 rpm rotor speed and a 0.5 mm sieve.

### 2.3. Proximate Composition of the Raw Materials

The protein content (total nitrogen × 6.25) was determined by using the Kjeldahl and Dumas methods according to the methods by the international organization for standardization (ISO) 8968-1:2014 and ISO 16634-2:2016, respectively. Ash, lipids, and moisture content were determined according to the methods ISO 8070:2007, ISO 1735:2004, and IDF26A:1993, respectively.

### 2.4. Characterisation of Oat Proteins

The oat proteins of destarched OPC samples were characterized with sodium dodecyl sulphate polyacrylamide gel electrophoresis (SDS-PAGE) fractionation using 10% Bis-Tris gel (Thermo Fisher Scientific, Invitrogen, Carlsbad, CA, USA), with non-reduced and reduced 10% 3-(N-Morpholino)propane sulfonic acid (MOPS) running buffer and the reducing agent was mercaptoethanol. SeeBlue Plus 2 Pre-stained Protein Standard (Thermo Fisher Scientific, Invitrogen, Carlsbad, CA, USA) was used as a molecular weight marker, and protein bands were stained with Coomassie Brilliant Blue.

### 2.5. Rheological Properties

The samples for rheological measurements were prepared by mixing protein materials with water (Table 1) for approximately 6 min at room temperature in a Farinograph (Brabender, Duisburg, Germany) with a 50 g mixing bowl. The mixed samples were packed in a sealed plastic bag and were stored at 8 °C until the samples were analyzed the following day. The samples were taken out from the cold storage 1 h before the rheological measurement. The rheological properties of protein materials were analyzed with a closed cavity rheometer (CCR) (APA2000, Alpha Technologies, Columbia, IN, USA). The samples (approximately 5.0 ± 0.5 g) were placed in the rheometer cavity between two plastic films. The plastic films were used to protect the CCR equipment. The cavity was bi-conical, where the lower cone was driven by a motor in an oscillatory movement and the upper cone was stationary. The cones had large grooves to prevent slippage and the sealed cavity was pressurized during measurements to prevent water evaporation. The height of the gap between the plates was between 0.48 and 3.0 mm from the center to the edges of the cones and the diameter was 44 mm.

Temperature sweep tests were performed following Emin et al. [19] with slight modifications. Temperature sweeps were done using temperatures between 60 and 160 °C (heating rate 5 °C/min), with a low strain of 1.5% and a low frequency of 1 Hz. After reaching 160 °C, the samples were cooled down to 30 °C by blowing air directly onto the sample. In the time sweep measurements, changes in the complex module G* as a function of time were followed for 10 min, and the temperature, strain, and frequency were kept constant at 140 °C, 80%, and 10 Hz, respectively. The parameters were chosen based on the method presented by Geerts et al. [22] with slight modifications based on the temperature sweep results. Rheological measurements were outside of the linear viscoelastic range (LVE) and therefore a complex modulus G* was reported. Each experiment was replicated three times.

### 2.6. High-Moisture Extrusion Cooking

Two extrusion mixtures were prepared, extrusion mixture 1 (EM 1), consisting of 60% PPI and 40% destarched OPC, and extrusion mixture 2 (EM 2, control sample), consisting of 100% PPI. The behavior of PPI in the high-moisture extrusion process was characterized in a previous study by Osen et al. [12] and was thus chosen as a control sample. The extrusion mixtures were blended with 50% water inside the extruder, which was calculated from the rate of water feed. The extrusion mixtures were extruded with a lab-scale high-moisture twin-screw extruder (Process 11 Hygienic, Thermo Scientific, Karlsruhe, Germany), equipped with a cooling die with dimensions of 4 × 20 × 250 mm (H × W × L). The process parameters were as follows: sample feed rate in the gravimetric sample feeder, 300 g/h; water feed rate, 330 mL/h; and screw speed, 300 rpm. The diameter and the length of the screws were 11 mm and 48 cm (L/D of the screws was 1:40). A more detailed description of the configuration of the screws is presented in the study by Nyyssölä et al. [23]. The barrel of the extruder consisted of eight heating blocks; the temperature of the first to the fifth block was kept constant (50, 60, 90, 120, and 130 °C). The temperatures from the sixth and seventh heating blocks were changed from 140 to 170 °C to study how the structure of the extruded product changed as a function of temperature. The temperature of the final heating block was kept constant at 115 °C. The temperature of the cooling die was 30 °C. Other parameters, such as torque (Nm), die pressure (bar), and melt temperature before the cooling die (°C), were recorded when the extrusion process reached a steady state. Once the steady state was achieved the samples were collected in set temperatures (140, 150, 160, and 170 °C).

### 2.7. Characteristics of the Extrudates

#### 2.7.1. Textural Properties

The cutting strengths of the extrudates were analyzed with an Instron universal testing machine (Instron 33R 4456, High Wycombe, England) using the Allo-Kramer shear cell with cutting blades while using a 5 kN load cell and cross-head speed of 400 mm/min. The samples were cut to 60 mm slices. The sample width (20 mm) and height (5 mm) were constant due to the cooling die dimensions. The samples were placed in the Instron longitudinally and blades of the Allo-Kramer shear cell cut them transversally. Three replicate measurements were carried out for each sample.

#### 2.7.2. Protein–Protein Interactions of Extrudates

Protein–protein interactions of EM 1 were determined following the method of Pietch et al. [24] with slight modifications. The extrudates from the high-moisture extrusion experiment were dried in a freeze dryer (Christ, Alpha 1-4 LDplus, Osterode am Harz, Germany) for 48 h. The freeze-dried samples were ground with a Bamix mixer to a fine powder. The extractability of proteins from EM 1 was determined with three sodium phosphate buffer solutions (pH 6.9). The first buffer was 0.2 M sodium phosphate buffer (PB) and disrupted electrostatic interactions. The second buffer solution was 0.2 M PB with 17.3 mM sodium dodecyl sulfate (SDS) and 8 M urea to disrupt non-covalent interactions. The third buffer solution was 0.2 M PB with 17.3 mM SDS, 8 M urea, and 10 mM of the reducing agent dithiothreitol (DTT) to disrupt the covalent and non-covalent interactions. The following extraction was done similarly in all buffer solutions. Ground samples (200 mg) were dissolved into 20 mL of buffer solution. The samples were first mixed with a vortex mixer and then mixed for 1 h with a magnetic stirrer. After this, the samples were centrifugated at 4637× *g* for 1 h and supernatants were collected for analysis of the protein content. The extractable protein content from the first buffer solution was analyzed in duplicate using a Dumas (Leco Corporation, City, MI, USA) according to ISO 16634-2:2016. The extractable protein contents from the second and third buffer solutions were determined using a Bio-Rad DC protein assay (Bio-Rad, Hercules, CA, USA), according to the manufacturer’s instructions, and by analysis of the samples at 750 nm with a UV-Vis spectrophotometer (UV-1700 PharmaSpec, Shimadzu, Kyoto, Japan). Bovine serum albumin was used as a standard protein. Measurements were replicated three times with duplicates resulting in a total of six values per extrudate. The extractable protein is expressed as a percentage of the total protein content in the untreated freeze-dried sample.

### 2.8. Statistical Analysis

All results were expressed as means ± standard deviation of at least triplicate measurement, if not mentioned otherwise. One-way ANOVA was used for statistical analysis of cutting strength and extractable protein and this was followed by Tukey’s honestly significant difference (HSD) with *p* < 0.05. Statistical analysis was performed using Minitab Statistical Software v. 20.1.1 (Minitab, Inc., State College, PA, USA).

## 3. Results and Discussion

### 3.1. Proximate Composition of the Raw Materials

The proximate composition of the raw materials was determined, and the composition of the extrusion mixtures was calculated based on the composition presented in Table 2.

### 3.2. Effect of Destarching on Oat Proteins

**Figure 1:**
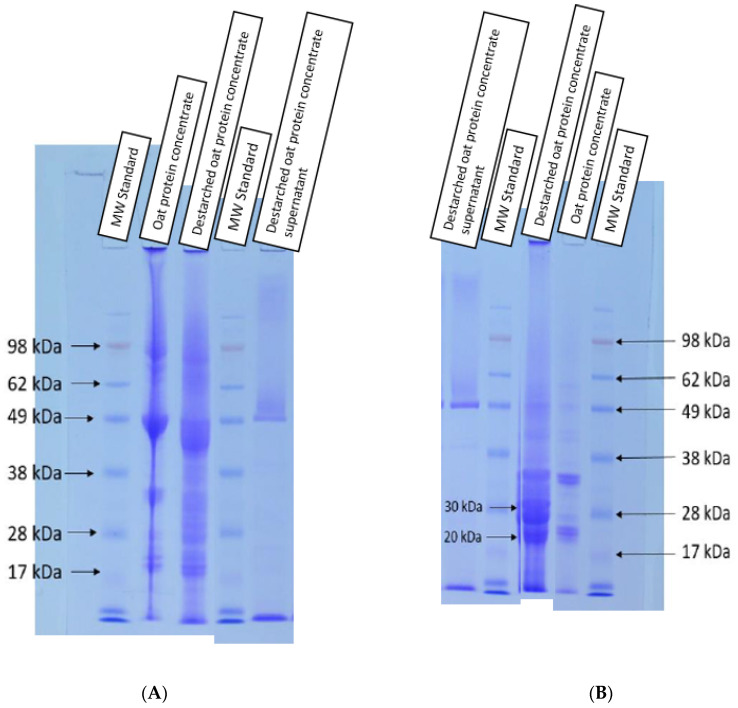
(**A**) Non-reduced 10% Bis-Tris gel and (**B**) mercaptoethanol-reduced samples of 10% Bis-Tris gel with indications of the sample molecular weight (MW standard), oat protein concentrate (starting material), destarched oat protein concentrate and destarched oat protein concentrate supernatant.

The SDS-PAGE was undertaken to determine whether the oat proteins were hydrolyzed during the destarching process due to the potential side activities of α-amylase. The main oat protein band was approximately 50 kDa in the non-reduced SDS-PAGE (Figure 1A) in both OPC and destarched OPC. Under reducing conditions, 30 kDa and 20 kDa bands were the most dominant. Walburg and Larkins [25] reported the 50 kDa band to be the native oat globulin protein, which breaks into 30 kDa and 20 kDa subunits in reducing conditions. The supernatant of destarched OPC did not contain any solubilized globulins, but there was a noticeable band at the 50 kDa area. This band was a rectangular shape. Mikola and Jones [26] reported that rectangular-shaped bands (similar to those in the supernatant of destarched OPC) at 50 kDa area are serpins (serinase protein inhibitors). Serpins do not usually show in the SDS-PAGE due to storage protein bands covering them. Inhibitors are small proteins, which specifically inhibit the actions of target enzymes. Based on the SDS-PAGE results, there were no noticeable changes in the protein structure.

### 3.3. Rheological Properties

**Figure 2:**
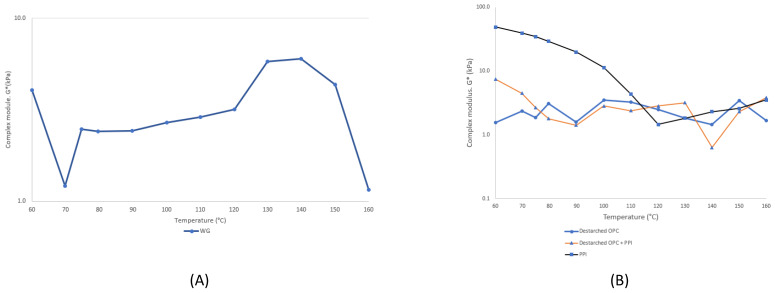
(**A**) The temperature sweep of wheat gluten (WG); (**B**) the temperature sweep of pea protein isolate (PPI) (control sample), destarched oat protein concentrate (OPC), and destarched oat protein concentrate + pea protein isolate. The temperature range was 60 to 160 °C and strain and frequency were kept constant (1.5% and 1 Hz, respectively).

The complex modulus G* of wheat gluten (WG) was determined with temperature sweep analysis by CCR to show that the gluten sample behaved similarly to the descriptions presented by Emin et al. [19]. Three regions can be recognized from the graph (Figure 2A). First there was a steep decline in the complex modulus (G*) at 70 °C, followed by an increase in the complex modulus as the temperature rose (80–120 °C), and peak values were achieved at higher temperatures (130–135 °C). The complex modulus decreased after 140 °C, which was due to the degradation of the WG structure [19].

With destarched OPC + PPI, the three areas where the complex modulus decreased and increased can be seen (Figure 2B). The first area of the G* decline was at 80–90 °C, there was a second small decline at 110 °C, and the third decline was observed at a higher temperature (140 °C). A similar decline in the complex modulus was observed in the sample with destarched OPC, which had a decline at 90 °C and another at 140 °C. In the control sample made from PPI, the complex modulus first declined continuously from 60 to 120 °C and then started to rise.

The decline of the complex modulus at 80 °C can be associated with the denaturation temperature of pea globulins [27]. There was a small decline in the complex modulus at 110 °C, which can be associated with the denaturation of oat globulin proteins [28]. Additionally, there was a small decline of the complex module at 140 °C. In the extrusion experiments (see Section 3.4), the changes in the melt temperature lower than 140 °C did not produce clear fibrillar structures in the end products. Moreover, using the blend of destarched OPC and PPI (EM1), a melt temperature of 140 °C or higher was needed to create fibrillar structures in the product. The same was observed in the case of EM2. When the melt temperature reached 120 °C, a fibrillar structure was observed. This corresponds with the decline of the complex module of the control PPI sample at 120 °C. A similar phenomenon has been reported previously. For example, Pietch et al. [24] utilized high-moisture extrusion to produce a fibrillar structure from WG at 155 °C and the melt temperature of WG was reported to be 130–135 °C [29]. These findings align with our WG temperature sweep results.

Quevedo et al. [30] investigated the denaturation and aggregation behavior of whey protein isolate (WPI) in extrusion-like conditions with CCR. They reported multiple regions (I–IV) where the complex modulus decreased and increased due to the protein denaturation and aggregation. Furthermore, onset denaturation and aggregation temperature can shift depending on factors such as protein concentration, water content, and shear rates due to the differences in denaturation kinetics [31]. For example, it has been reported that the onset denaturation temperature of WPI decreased from 73 to 66 °C when shear rates were increased from 0.06 to 50 s^−1^ [30]. The differences in protein concentration between the samples could explain the noticeable change in the behavior of the complex modulus of the PPI control sample compared to the other samples.

**Figure 3:**
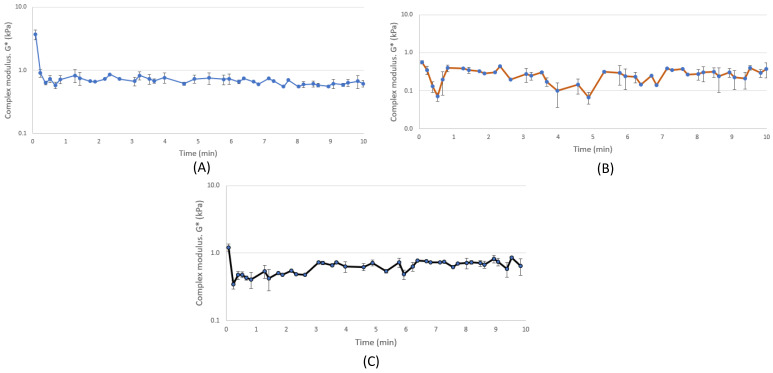
(**A**) Change in the complex modulus (G*) as a function of time of pea protein isolate; (**B**) change in the complex modulus (G*) as a function of time of destarched oat protein concentrate; (**C**) change in the complex modulus (G*) as a function of time of destarched oat protein concentrate + pea protein isolate. The temperature, strain, and frequency were kept constant in all of the measurements (140 °C, 80%, and 10 Hz, respectively). All measurements were carried out in triplicates except (**B**). The standard error bars in the graph are the standard errors of that specific point (*n* = 3, and *n* = 2 for (**B**)).

The time sweep measurements were carried out at 140 °C with a high strain (80%) and high frequency (10 Hz) for 10 min. The conditions in the time sweep measurements were set to mimic the process applied in the high-moisture extrusion at constant temperature. At the beginning of the time sweep graphs (Figure 3), there was a noticeable decline and rise in the complex modulus. This might have been due to the denaturation and aggregation of proteins [22]. The sample with destarched OPC + PPI showed an increase of G* as a function of time (Figure 3C). The complex modulus G* increased slowly (3 min) and then reached a steady state. This type of behavior was not observed in the time sweep graphs of the samples for PPI or destarched OPC alone. The sample with destarched OPC (Figure 2B) had more deviation compared to the other samples because of the huge variance between measured data points. The reason might have been that measurement was close to the detection limit of the CCR [20]. In previous studies, a similar type of behavior was reported in the complex modulus with soy protein and the blend with soy protein and wheat gluten, which were analyzed with a CCR with similar parameters [22,32]. Pietch et al. [11] suggested that this behavior was explained by the polymerization or aggregation of proteins as a function of time.

### 3.4. Structure Properties of the Extrudates

The ratio of PPI and destarched OPC in EM1 deviated from the blend that was used in the CCR tests. It was presumed that the CCR blend had too low a protein concentration to form any fibrillar structure in the high-moisture extrusion. This presumption was based on earlier reports that recommend that, to achieve fibrillar structures, the protein concentration should be between 50–70% [15,33]. For example, Angelis et al. [15] reported that suitable protein concentrations for producing a meat analog from dry-fractionated pea protein concentrate and commercial OPC should be above 50%.

**Figure 4:**
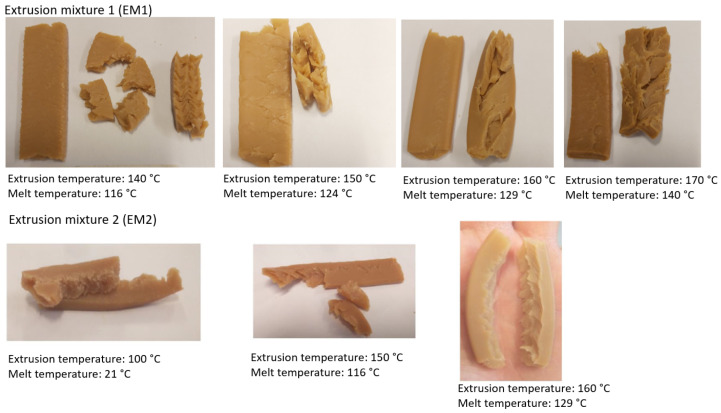
Formation of the fibrillar structure of extruded samples (EM1 and EM2).

Fibrous structures were successfully produced by high-moisture extrusion from EM1 and EM2 temperatures ranging from 160 to 170 °C (Figure 4). The control sample EM2 first exhibited a dough-like structure at lower temperatures (120–150 °C). When the extrusion temperature (>150 °C) and the melt temperature (>120 °C) increased, a noticeable formation of a fibrous structure with a smooth surface was achieved. In addition, Osen et al. [12] reported that the macrostructure of an extruded fibrillar pea protein isolate product at 160 °C had a homogeneous structure with a smooth surface. Grabowska et al. [34] demonstrated that when soy protein concentrate was processed with a high shear cell, it did not form a fibrous structure at 120 °C, but when the temperature was raised to 140 °C, a layered structure with thick fibers was observed. The EM1 required higher temperatures (170 °C) and higher melting temperatures (140 °C) than EM2 to form similar fibrous structures. It can be concluded that the temperature and the melting temperature had a noticeable effect on the fibrillar structure of the extruded samples. Additionally, a fibrillar structure from EM1 was successfully formed despite the low protein content (30%).

When measuring the cutting strength with the Allo-Kramer shear cell, the orientation of fibers is an important factor, which can lead to inaccurate results. It has been demonstrated by Bouton et al. [35] that, in order to measure cutting strength from chicken, the samples need to line up so that the blades cut perpendicularly through the muscle fibers.

**Figure 5:**
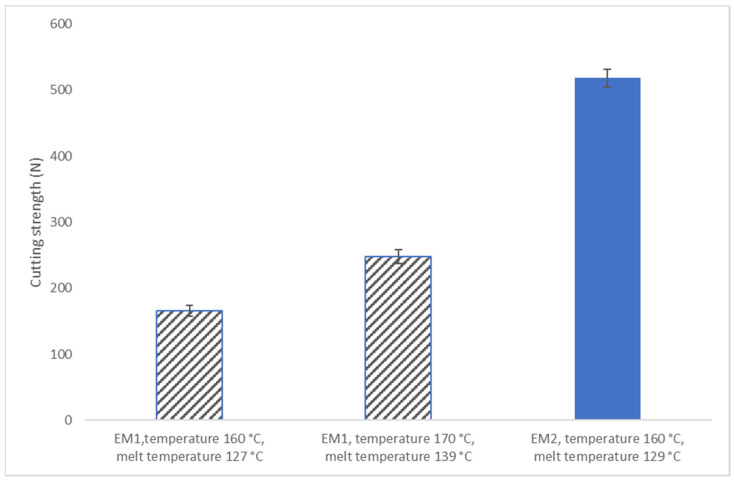
The cutting strength of the extrusion mixture 1 (EM1) (melt temperatures: 127 and 139 °C) and extrusion mixture 2 (EM2) (melt temperature: 129 °C) samples. The samples were placed longitudinally and blades of the Allo-Kramer shear cell cut them transversally.

The cutting strength increased in the EM1 sample from about 200 to 250 N when the melt temperature increased from about 130 to 140 °C (Figure 5). Kennedy et al. [36] reported a similar increase in the cutting strength of extruded decorticated cowpea meal. They reported that, at the moisture content of 40%, the cutting strength increased from 259 to 342 N when the extrusion temperature was increased from 150 to 200 °C. Furthermore, the addition of destarched OPC decreased the cutting strength when compared to the extruded sample made from PPI (EM2). The cutting strength of cooked broiler *Pectoralis major* muscle considered tender or very tender has been reported to be 110 N and patty made from a ground chicken file was 40 N [37,38]. The cutting strength of extrudates was noticeable higher compared to the firmness of the chicken breast. High cutting strength values for textured vegetable protein products have been previously reported by Saio [39]. He investigated the cutting strength of commercial textured or fiberized vegetable protein products that were made from soy protein or wheat gluten. He reported that the mean cutting strength value of texturized soybean products (sample size was 54) was 927 N and the mean cutting strength value of wheat gluten products (sample size was 24) was 538 N.

#### Effect of Extrusion Process on the Protein–Protein Interactions

**Figure 6:**
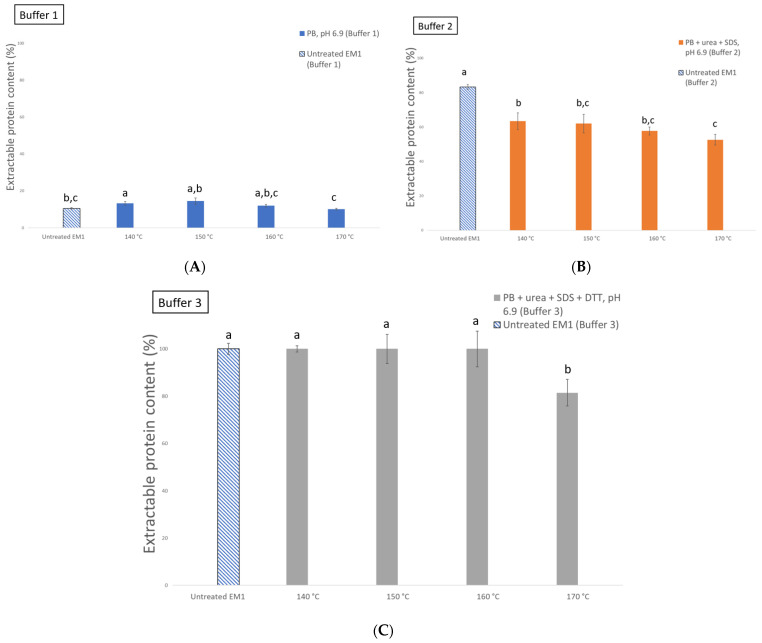
Change in the extractable protein content from the untreated destarched oat protein concentrate + pea protein isolate flour blend and the freeze-dried extrusion mixture 1 (EM1) at different extruder temperatures (140–170 °C): (**A**) after the disruption of electrostatic interactions (buffer 1); (**B**) non-covalent interactions consisting of electrostatic, mostly hydrophobic, and hydrogen bonds (buffer 2); (**C**) non-covalent and covalent interactions (buffer 3). Different letters (a, b, c) indicate significant differences (*p* < 0.05) within the buffer group (buffer 1–buffer 3).

The dominant protein–protein interactions of freeze-dried EM1 extrudates at different extrusion temperatures ranging from 140 to 170 °C were determined by using buffer solutions to solubilize the proteins. The amount of extractable protein was significantly (*p* < 0.05) different between the selective buffers. The disruption of the weaker electrostatic interactions with PB buffer (buffer 1) could release only <20% of the proteins (Figure 6A). The extractable protein content was increased from about 10 to 60% when non-covalent bonds (electrostatic, hydrophobic, and hydrogen bonds) were disrupted with PB + urea + SDS (buffer 2) (Figure 6B). The solubility increased to 100% when the freeze-dried extrudates were treated with PB + urea + SDS + DTT (buffer 3), which disrupted non-covalent and covalent interactions (Figure 6C). These results are in accordance with previous studies that have shown that non-covalent and covalent bonds are responsible for the low solubility of proteins in the extrudate samples of buffers with the same composition as buffer 1 [18,40,41]. Furthermore, it has been suggested that non-covalent bonds, such as hydrophobic interactions and hydrogen bonds, as well as covalent disulfide bonds play a major role in the formation and stabilization of the rigid structures of extrudates produced with high-moisture extrusion [18,42,43]. Additionally, the protein extractability decreased significantly (*p* < 0.05) in buffer 2 when the extrusion temperature was raised from 140 to 170 °C compared to the untreated sample. However, the protein extractability decreased significantly (*p* < 0.05) in buffer 3 only at 170 °C. This suggests that there was a decrease in non-covalent and covalent bonds due to the formation of insoluble protein complexes.

Ottenburn et al. [44] have demonstrated the formation of isopeptides at elevated processing temperatures. Isopeptides are a formation of heat-induced crosslinks between aspartyl, glutamyl, and lysinyl residues. These chemical and enzymatically resistant crosslinks decrease the extractability of proteins in selective buffers [45]. It has been reported by Hurrel and Carpenter [46] that the formation of isopeptide bonds through heating reduced the nitrogen and lysine digestibility by up to 20% due to the blocking of the enzyme active sites. Hellwig et al. [47] demonstrated in an in vitro study that γ-glutamyl-ε-lysine isopeptides had low digestibility. However, in vivo studies showed the opposite results, demonstrating that rats were able to digest and absorb the lysine bound in the glutamyl-lysine isopeptides [46,48]. Rather than glutamyl-lysine, which had full bioavailability, β-aspartyl-ε-lysine appeared to be the reason for the decreased protein digestibility [49].

Extrusion at high temperatures has been shown to decrease bioavailability of certain amino acids and decrease protein digestibility [50]. For example, extrusion undertaken with low or moderate moisture contents (15–30%) at temperatures from 160 to 170 °C has been shown to decrease the lysine bioavailability in soy proteins [50]. On the other hand, a study by MacDonald et al. [51] on the growth of rats showed that high-moisture extrusion preserved the bioavailability of lysine. On the other hand, the opposite results have also been found, showing that extrusion improved the digestibility of pulse proteins [52]. For example, Palanisamy et al. [52] showed that lupin proteins extruded at temperatures from 135 to 180 °C and at moisture contents from 40 to 55% had 3 to 6% higher digestibility compared to the untreated raw material. Furthermore, Duque-Estrada et al. [53] studied a soy protein meat analog that was made with a high-temperature shear cell at temperatures of 100 to 140 °C. They reported that the in vitro protein digestibility of soy proteins did not decrease despite the decrease in the protein solubility and the higher level of carbonyls in the protein due to the thermomechanical treatment. The possible increase in protein digestibility has been hypothesized to be caused by the unfolding of major globulins and the inactivation of anti-nutrients, such as trypsin inhibitors [50].

## 4. Conclusions

In this work, enzymatically hydrolyzed destarched OPC was successfully utilized in high-moisture extrusion together with pea protein to create a meat analog with a fibrillar structure. This provides a novel product concept for the growing meat analog sector. The enzyme treatment utilized in this work to produce the destarched oat protein concentrate mimicked the enzyme treatment applied in the production of oat milk. The destarched oat protein concentrate corresponded to the side-stream that is created during the removal of the insoluble fraction from the enzymatically hydrolyzed oat suspension. The method presented in this work could be potentially deployed to utilize this insoluble oat fraction to create novel meat analogs together with other protein sources, such as pea protein. The CCR was successfully utilized in the characterization of the denaturation and aggregation behavior of destarched OPC, PPI, and their blend. In the extrusion experiments for both extrusion mixtures, clear fibrillar structures were achieved at temperatures that were above the highest temperature for which denaturation and aggregation were recorded in the temperature sweep data. This observation raises an interesting question concerning whether a CCR be used to determine the required melting temperature for which the formation of fibrillar structures would be achieved with high-moisture extrusion. Additionally, the protein extractability was significantly decreased at higher extrusion temperatures. It has been shown that isopeptides form at high temperature conditions, which reduces protein digestibility and decreases the bioavailability of certain amino acids such as lysine. Furthermore, traditional oat protein ingredients need pre-treatments before they can be utilized in industrial processes. There is a need for further studies on how to improve the functionality of oat proteins to produce highly functional plant-based protein ingredients for the food industry.

## Figures and Tables

**Table 1:** The measured ratios of destarched oat protein concentrate, pea protein isolate, wheat gluten, and destarched oat protein concentrate with pea protein isolate for the closed cavity rheometer measurements.

Sample	Protein Powders (%)	Water (%)
Wheat gluten	55	45
Destarched oat protein concentrate	55	45
Pea protein isolate	55	45
Destarched oat protein concentrate + pea protein isolate	30 + 25	45

**Table 2:** The chemical composition of the commercial raw materials, destarched oat protein concentrate, and a blend of these proteins. The calculated proximate compositions of the extrusion mixtures EM1 (destarched OPC + PPI) and EM2 (PPI) are shown.

Sample	Protein (wt%)	Fat (wt%)	Ash (wt%)	Moisture (wt%)
Oat protein concentrate	25.6 ± 0.0	4.9 ± 0.2	4.0 ± 0.0	7.32 ± 0.0
Pea protein isolate	77.5 ± 0.0	8.9 ± 0.2	4.5 ± 0.0	6.9 ± 0.1
Destarched oat protein concentrate	33.1 ± 0.4	6.79 ± 0.0	5.1 ± 0.0	11.6 ± 0.0
Pea protein isolate + destarched oat protein concentrate blend	60.0 ± 0.3	7.61 ± 0.1	5.0 ± 0.0	7.6 ± 0.1
EM 1 *	30	3.8	2.5	63.7
EM 2 (control) *	38.8	4.5	2.3	54.4

* Calculated from results based on PPI + destarched OPC mix and PPI, respectively.
